# Prognostic value of *p53* for colorectal cancer after surgical resection of pulmonary metastases

**DOI:** 10.1186/s12957-016-1049-4

**Published:** 2016-12-21

**Authors:** Cong Li, Qi Xu, Lei Chen, Cong Luo, Yinbo Chen, Jieer Ying

**Affiliations:** Zhejiang Cancer Hospital, No. 1 Banshan East Road, Gongshu District, Hangzhou, Zhejiang China

**Keywords:** Prognostic value, *p53*, Colorectal cancer, Lung metastasis, Surgical resection

## Abstract

**Background:**

Pulmonary metastases occur in up to 25% of colorectal cancer (CRC) patients. Many studies have reported that pulmonary metastasectomy might increase 5-year survival of these patients. The aim of this study was to describe our experience with pulmonary metastasectomy for metastatic colorectal cancer and to explore the prognostic value of *p53* overexpression and other factors.

**Methods:**

Between July 2002 and December 2013, the clinicopathological data of 88 patients with colorectal carcinoma who underwent pulmonary metastases resection were retrospectively reviewed and analyzed. Clinical, biochemical and imaging, and operative data, and expression of *p53* were retrospectively collected. Immunohistochemical staining for *p53* was performed on paraffin-embedded 5-μm sections using mouse anti-human tumor protein *p53* monoclonal antibody (DO-7, Dako, Denmark). Overall survival (OS) was calculated from resection of pulmonary metastases to death. The prognostic effect of each variable on survival was evaluated using the Kaplan-Meier method and log-rank test. For the multivariate analysis of prognostic factors, the Cox regression model was used.

**Results:**

There were 58 men and 30 women in this study, and their median age was 55 (range 31 to 85). Video-assisted thoracoscopic surgery (VATS) was performed in 59 cases (78%), and 29 patients (19%) underwent thoracotomy. Lung wedge resection and pulmonary lobectomy were performed in 52 (59.1%) and 36 (40.9%) patients, respectively. After a median follow-up duration of 44 months, the cumulative 5-year survival was 45.4%, and the median overall survival was 57.8 months. The expression of *p53* significantly influenced survival. In patients with *p53* protein overexpression, we observed a median OS of 46.1 months, whereas the median OS of patients with negative protein expression of *p53* was 62.6 months (*p* = 0.047). However, in multivariate analysis, *p53* overexpression was failed to be an independently significant prognostic factor for survival.

**Conclusions:**

Pulmonary resection of metastatic colorectal cancer might offer a chance to prolong survival including those patients with extrapulmonary metastases. *p53* protein expression was identified as a prognosis-related factor for surgery.

## Background

Colorectal cancer (CRC) is the third most common malignancy all round the world [1]. At least 50% of CRC patients will develop a metastatic disease and about 5–25% of them are located in the lung [2]. Due to the development of new chemotherapeutic drugs and minimal invasive video-assisted thoracoscopy and the fact that liver metastasectomy contributes to survival improvement, pulmonary metastasectomy has emerged as a potentially curative option in the multimodal management of metastatic CRC [2, 3]. When compared with 5% for patients without pulmonary metastasis local treatment, the reported 5-year survival of CRC can be elevated up to 62% after pulmonary metastasectomy [4]. Since the 1990s, a large number of retrospective studies have shown that multiple clinical features may be probable prognostic survival factors for patients after metastasectomy, such as the number of metastases, preoperative carcinoembryonic antigen serum (CEA) level, thoracic lymph node involvement, and surgical procedures [4–8]. However, reliable and objective prognostic variables are still in urgent need of distinguishing patients who benefit from a surgical approach [2, 4]. As a common tumor suppressor, *p53* overexpression is thought to be a probable prognostic factor and response to therapy. Nevertheless, the prognostic value of *p53* overexpression in CRC patients after pulmonary metastasectomy was investigated in few studies. Therefore, the objective of this retrospective study was to describe our experience in pulmonary metastasectomy for metastatic CRC and explore whether *p53* overexpression has prognostic value in pulmonary metastasectomy of CRC.

## Methods

Between July 2002 and December 2013, 88 patients underwent resection of pulmonary metastases from colorectal cancer. Each patient had a proven tissue diagnosis of metastatic colorectal adenocarcinoma. The criteria for resection of pulmonary metastases included unilateral or bilateral resectable lung lesions, no local recurrence of primary lesions, and adequate cardiorespiratory function for complete resection of all pulmonary lesions. Extrapulmonary metastases of light tumor burden were included. Clinical, biochemical and imaging, and operative data were collected from computerized records. Immunohistochemical (IHC) staining for *p53* was performed on paraffin-embedded 5-μm sections using mouse anti-human tumor protein *p53* monoclonal antibody (DO-7, Dako, Denmark). Samples were considered positive when at least 20% of the cancer cells were positive for *p53* staining. Follow-up data were obtained from the patients’ records and by contacting the patients’ respective general practitioners. The data were analyzed by SPSS 13.0 version. The prognostic effect of each variable on survival was evaluated using the Kaplan-Meier method and log-rank test. For the multivariate analysis of prognostic factors, the Cox regression model was used. *P* value of less than 0.05 was considered statistically significant.

## Results

### Patient characteristics

The study included 58 men (65.9%) and 30 women (34.1%), and the median age was 55 years (range 31 to 85 years). The primary tumor location included 25 (28.4%) in the colon and 63 (71.6%) in the rectum. Pulmonary metastasis was solitary in 71 patients (80.7%) and multiple in 17 patients (19.3%, unilateral in 5, bilateral in 12). Five patients had extrapulmonary metastases, including two solitary liver metastases (one of them had liver metastases resection after pulmonary metastasectomy), one thoracic lymph node metastasis, one pelvic soft tissue metastasis, and one pleura metastasis, and none of them received treatment for metastasis. Fifty-nine patients (67%) underwent palliative chemotherapy, and the common regimens were irinotecan-based or oxaliplatin-based combined chemotherapy. Among them, 24 patients (27.3%) underwent more than one line chemotherapy. Video-assisted thoracoscopic surgery (VATS) was performed in 59 cases (78%) and thoracotomy in 29 patients (19%). Fifty-two patients (59.1%) underwent lung wedge resection, and 36 patients (40.9%) underwent pulmonary lobectomy (Table 1).

**Table 1:** Clinical and pathological characteristics of 88 patients and univariate analysis for OS

Factor	Number (%)	OS (months)	*p* value
Age (years)			
≤60	61 (69.3)	57.8	0.475
>60	27 (30.7)	55.6	
Gender			
Male	58 (65.9)	57.8	0.729
Female	30 (34.1)	67.9	
Tumor size of lung metastasis (cm)			
≤3	75 (85.2)	62.6	0.212
>3	13 (14.8)	47.2	
Primary tumor localization			
Left-sided colon	9 (10.2)	41.2	0.185
Right-sided colon	16 (18.2)	67.9	
Rectum	63 (71.6)	61.6	
Metastases phase			
Synchronous metastases	10 (11.4)	46.1	0.661
Metachronous metastases	78 (88.6)	57.8	
Pulmonary metastases number			
Single	71 (80.7)	62.6	0.23
Multiple	17 (19.3)	49.8	
Extrapulmonary metastasis			
Yes	5 (5.7)	39.5	0.756
No	83 (94.3)	57.8	
Surgical procedures			
VATS	59 (67.0)	62.6	0.516
Thoracotomy	29 (33.0)	57.8	
Resection range			
Lung wedge resection	52 (59.1)	67.9	0.696
Pulmonary lobectomy	36 (40.9)	57.8	
Preoperative CEA level			
Normal	64 (72.7)	62.6	0.142
Elevated	24 (27.3)	42	
*p53* IHC			
Positive	42 (47.7)	46.1	0.047
Negative	46 (52.3)	62.6	

*IHC* immunological histological chemistry, *CEA* carcinoembryonic antigen

We also analyzed the correlation between *p53* expression level and clinicopathological factors, but none of them has significant relationship with *p53* status (Table 2).

**Table 2:** Relationship between *p53* protein levels in colorectal tumors and clinicopathological variables

Variable	Number (%)	*p53* positive	*p53* negative	*p* value
		(*n* = 42)	(*n* = 46)	
Age	Range	35–76	31–85	–
	Median	53	55	
Sex	Male	29	29	0.654
	Female	13	17	
T status	T1-2	9	8	0.788
	T3-4	33	38	
N status	N0	17	27	0.135
	N+	25	19	
Site	Left colon	5	4	0.851
	Right colon	7	9	
	Rectum	30	33	
Preoperative CEA	<5ng/ml	28	36	0.241
	>5ng/ml	14	10	

### Survival analysis

Median follow-up duration was 44 months (range 3 to 162 months). The median interval between CRC diagnosis and lung metastasis diagnosis was 25 months (range 0 to 122 months). After pulmonary metastasectomy, disease recurrence was identified in 26 of the 88 patients (distant metastasis in 7, local recurrence in 19). The interval between pulmonary metastasectomy and disease recurrence had a median of 13.5 months (range 4.2 to 34.8 months). At the last follow-up, 51 patients (58.0%) were alive and the cumulative 5-year survival was 45.4%, and the median survival was 57.8 months.

**Fig. 1:**
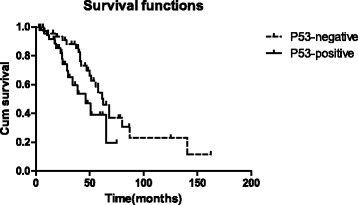
Probability of survival of patients with positive expression of *p*53 vs patients with negative expression (*p* = 0.047)

Among the analyzed prognostic factors, age, gender, maximum tumor size of lung metastasis, localization of the primary tumor, metastases phase, and the number of lung metastasis, extrapulmonary metastases and preoperative CEA level did not influence survival significantly. However, *p53* overexpression greatly affected survival. In patients with negative *p53* expression, an overall survival of 62.6 months was observed, whereas an overall survival of 46.1 months was observed in patients with positive *p53* expression (*p* = 0.047) (Table 1, Fig. 1). Furthermore, repeated analysis in patients with solitary or multiple lung metastases showed the prognostic value of *p53* protein expression. However, *p53* was not confirmed to be an independently significant prognostic factor for survival in multivariate analysis (*p* = 0.051).

## Discussion

Even though there is no randomized trial evidence, resection of pulmonary metastases is considered to be an effective measure to improve the survival of the appropriately selected CRC patients with lung metastases. Multiple studies have evaluated a number of possible clinical or pathological prognostic indicators. However, the relationship between molecular abnormalities and survival is unclear [5, 9–12].

As a tumor suppressor gene, *p53* is a common target of genetic alteration in human cancer [13, 14], which exerts the function of controlling the induction of growth arrest and apoptosis by eliminating damaged cells [15, 16]. Alteration of this gene is associated with postoperative outcome and poor prognosis [17]. Technically, *p*53 protein expression can be assessed by IHC and *p*53 genomic status can be analyzed using direct gene sequencing [18]. In consideration of its technical reproducibility and high concordance with that of genomic analysis, IHC still holds considerable promise as a convenient and inexpensive means [18]. Therefore, IHC was adopted as a method to assess the *p*53 expression in this study.

Previous studies have revealed *p*53 protein expression with the help of IHC in 42–69% of CRCs [15, 16, 18]. Multiple tumors with increased *p*53 expression were associated with lymph node metastasis, extrathyroidal invasion, pleural infiltration, and tumor location [19, 20]. Literature review showed that *p*53 overexpression could be an adverse independent predictor of survival [21, 22]. In addition, *p*53 expression was found to be related to liver metastases of colorectal tumors in some studies [23–25]. However, some investigations failed to document a significant inverse correlation of *p*53 overexpression with CRC patients’ survival after pulmonary metastasectomy. One possible reason was that there were so many variables related to staining protocols and scoring systems that contrasting results were provided [26]. More importantly, the relationship between *p53* overexpression and gene alteration was actually complicated [27]. Protein overexpression is seen in gene mutation type and cases with wild type [26, 28]. Generally, different kinds of antibodies mainly recognize certain mutants or wild type of genes, which cannot fully reflect protein expression to some extent.

In this study, *p53* overexpression was shown in 42 (47.7%) cases with a significantly shorter survival (median OS 62.6 vs 42.1 months, *p* = 0.046), which suggested a potential candidate for modulating the risk of colorectal lung metastases of *p53*. However, a single-center retrospective design and highly selected patient population could have introduced biased information. Additionally, the small size of the population and the co-linear relation between the parameters may give rise to meaningless results of multivariate analysis. Therefore, a larger trial is needed to confirm the value of *p53* overexpression or mutation.

## Conclusions

In conclusion, surgical resection of pulmonary metastases may extend the survival of CRC patients even if there are extrapulmonary metastases. Besides, *p53* overexpression may help judge the prognosis of patients who undergo pulmonary metastasectomy.

## Abbreviations

CEA: Carcinoembryonic antigen serum
CRC: Colorectal cancer
IHC: Immunohistochemical
VATS: Video-assisted thoracoscopic surgery
